# Severe acute pancreatitis – a serious complication of leptospirosis

**Published:** 2013-09-25

**Authors:** D Popa, D Vasile, A Ilco

**Affiliations:** "Carol Davila" University of Medicine and Pharmacy, Department of Surgery, Bucharest University Emergency Hospital, Bucharest, Romania

**Keywords:** Leptospirosis, Acute Pancreatitis

## Abstract

Leptospirosis is a disease caused by pathogenic spirochetes of genus Leptospira. It is considered the most common zoonosis in the world. Acute pancreatitis is a rare complication of leptospirosis (25%). We present the case of a 34-year-old male patient with severe leptospirosis complicated with acute renal failure. After 9 days from the onset of the disease, the patient developed acute necrotizing pancreatitis, infected from the very beginning, associated with multiple organ failure, septic shock and severe anemia. The diagnosis was clinically and biologically stated and confirmed by CT-scan. The patient underwent surgery for infected necrotizing acute pancreatitis of the head and neck of the pancreas, with left retroperitoneal expansion down to the left iliac fossa. We performed a necrosectomy with the evacuation of the tisular debris, multiple drainage of the peritoneal cavity, followed by an open abdomen with synthetic mesh. The postoperative evolution was difficult but constantly progressive. Two reinterventions were necessary. The patient left the hospital in good condition after 75 days postoperatively.

## Introduction

Leptospirosis was first mentioned in 1812 by Larrey and is commonly known as the "yellow fever". It is the most frequent zoonosis in the world caused by the pathogenic spirochetes from leptospira family [**1**].

 This disease is characterized by two clinical forms:

 - mild (easy-medium): represents 90% of cases

 - severe 10% of cases. Adolph Weil first described this form; therefore, it is also called the Weil disease or icterohaemorrhagiae leptospirosis (IHL).

 The complications of icterohaemorrhagiae leptospirosis (IHL) [**2**] are represented by: acute renal failure (95% of cases), acute hepatic failure (72% of cases), acute respiratory failure (38% of cases), acute cardiovascular failure (33% of cases) and acute pancreatitis (25%).

## Material and methods

The paper is a case presentation of a patient admitted and operated within the First Surgery Clinic of the University Emergency Hospital Bucharest. 

 We present the case of a male patient, 34 years old, diagnosed with IHL on 13/09/2009 within “Prof Dr Matei Bals" Institute of Infectious Diseases in Bucharest. On 16/09, he was transferred to the Nephrology Clinic in the University Emergency Hospital due to acute renal failure (ARF). After nine days, on September 25, he was transferred to the First Surgery Clinic in the same hospital with the following diagnosis: 

 - acute necrotizing pancreatitis – puss collection in the lesser sack and retroperitoneal space

 - icterohaemorrhagiae leptospirosis (IHL) with acute renal failure (ARF).

 - severe jaundice

 - severe anemia (Hb=4g/dl)

 - multiple organ failure (MOF)

 - sepsis

 Clinical diagnosis: the general condition of the patients was severely altered with dyspnea, moderate jaundice, extreme paleness, fever (39°C), abdominal pain, nausea and vomiting. 

 The local examination revealed abdominal distension, with discrete mobility following breathing with the evidence of a 12-15 cm diameter prominence in the superior abdomen. At this level, we could find on palpation a diffuse delimitated painless tumor mass of about 10-15 cm in diameter. On the left abdominal flank and left iliac fossa, we could feel a diffuse delimitated area with induration, which was intensively painful. The epigastric tumoral surface was dullness to percussion presenting signs of peritoneal irritation. Under-umbilical abdominal area was characterized by hyper-sonority. 

 Imaging diagnosis

 Abdominal ultrasound: 

 16/09: steatosis liver, peritoneal ascites in Morrison space, homogeneous pancreas with cephalic diameter of 20 mm and liquid in the Douglas pouch

 23/09: pancreas with cephalic diameter of 32 mm, diffuse non-homogeneously and hyper-echoic structure. Anterior from the body and tail of the pancreas and posterior to the stomach we could identify a mixed structure, predominant transonic, irregular, which was fading the pancreatic shape, having a size of 70 mm in diameter; this image also presented an extension towards the visceral face of the spleen.

 C.T. scan: 

 25/09: diffuse enlargement of the pancreas with necrosis associated with necrotic collections in the lesser sack, anterior and inferior to the pancreas tail; necrotic retroperitoneal extensions, anterior to the left pre-renal fascia and also behind the descendent colon.

 Abdominal X-ray 

 17/09: hidroaeric levels of the small bowel in the center of the abdomen. Thoracic X-ray and the gastroscopy were normal.

**Fig. 1:**
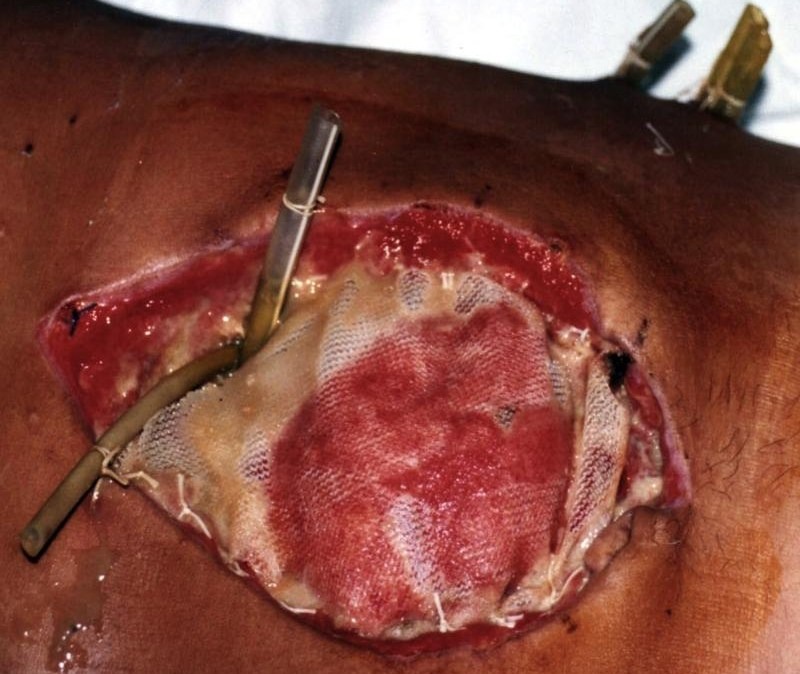
Open abdomen with mesh

 The decision was surgical intervention on September 25 and the following were also performed: explorative laparotomy, entering through the gastro-colic ligament into the lesser sack and evacuation of the cephalic- corporeal abscess; also the incision and evacuation of retroperitoneal extensions, abdominal extensive lavage and multiple drainage. Finally, open abdomen with mash (**Fig. 1**).

**Fig. 2:**
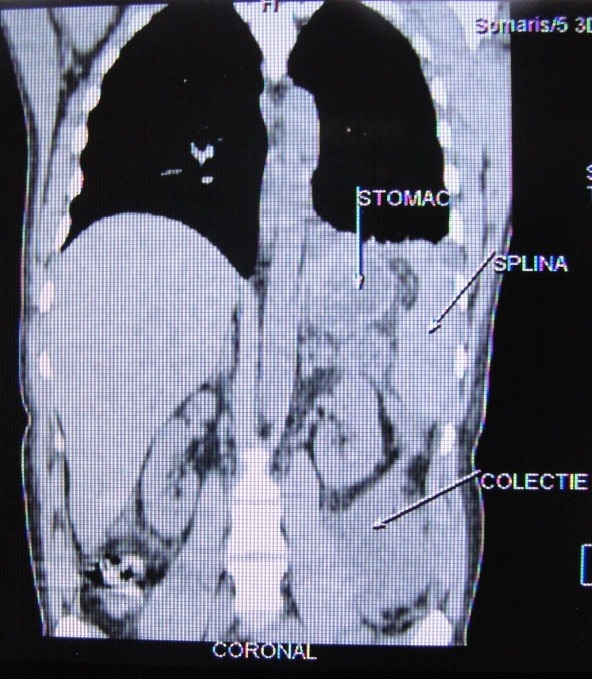
CT scan showing retroperitoneal necrotic extensions

The general condition was stationary; this is why a new intervention was decided on October 6 (**Fig. 2**).

We performed the following: reexploratory laparotomy, removing the mesh, reevacuation and drainage of the retroperitoneal extensions, extensive lavage and drainage of the abdominal cavity.

 After this second operation, the general condition improved slowly (52 days), the patient became hematologically stable; the renal, hepatic and respiratory failure disappeared. Digestive tolerance and bowel peristaltic were also regained.

 On October 29, a third and final operation was performed during which we controlled and drained the left retroperitoneal extension, the left colic-parietal space and the right extremity of the transverse mesocolon root. In addition, complex adhesiolysis was completed and a single layer suture closed the abdomen. 

 Despite the favorable local and general evolution, the patient still presented fever almost continuously associated with persistent leukocytosis.

On November 11, the test for leptospirosis was positive, showing the necessity of a continuous etiologic treatment. 

 On 17/11he was transferred to “Dr. Matei Bals" Infectious Diseases Institute, in sepsis state, with the following diagnose: Staf Aureus Meticilinoresistant sepsis, having the origin in a pancreatic abscess after being infected with acute necrotizing pancreatitis, due to a severe form of icterohemorrhagic leptospirosis (IHL). Antibiotherapy was associated for 28 days. 

 Tienam 3g/day

 Targocyd 400 mg/day

 Moxifloxacin 1 tb/day

 Rifampicin 600 mg/day

 Two CT exams were performed, on 21/11 and 12/12, that reflected local favorable evolution, evident diminishing of the pancreas dimensions, the minimum infiltration of adipose plans showing a favorable local and regional evolution, an obvious decrease in the size of the pancreas, slightly infiltration in the peripancreatic cleavage adipose plans and the disappearance of the retroperitoneal expansion fuse. 

 On 19/12, (approximately 3 months post operatory): the patient was cured.

 The leptospirosis test was negative (05/01).

## Conclusion

1) Severe form of IHL is a serious disease with a mortality rate of de 30% [**2,3**].

2) Acute Pancreatitis (A.P.) is a rare complication of IHL – 25% [**2,4,5**].

3) Precocious diagnosis is essential for IHL treatment as for A.P. treatment.

4) Regularly, A.P. caused by IHL is of medium severity [**6,7**] and responds well to medical treatment [**6,8,9**].

5) Severe A.P. that occurs in case of IHL is rare and with a rapid evolution towards infected necrosis (as in the case we presented).
